# Antimicrobial resistance national level dialogue and action in Ghana: setting and sustaining the agenda and outcomes

**DOI:** 10.1186/s42522-021-00051-w

**Published:** 2021-10-19

**Authors:** Augustina Koduah, Martha Gyansa-Lutterodt, George Kwesi Hedidor, Reginald Sekyi-Brown, Michelle Asiedu-Danso, Brian Adu Asare, Angela Ama Ackon, Edith Andrews Annan

**Affiliations:** 1 Department of Pharmacy Practice and Clinical Pharmacy, School of Pharmacy University of Ghana, P. O. Box LG43, Legon, Ghana; 2 Technical Coordination Directorate, Ministry of Health, P. O. Box M44, Accra, Ghana; 3 World Health Organization Country Office for Ghana, P.O Box MB 142, Accra, Ghana; 4 Pharmacy Directorate, Ministry of Health, P. O. Box M44, Accra, Ghana; 5 World Health Organization African Region, P.O. Box CY 348, Causeway, Harare, Zimbabwe

**Keywords:** Agenda setting, Antimicrobial resistance, Ghana, Multisectoral coordination, One health approach

## Abstract

**Background:**

Antimicrobial resistance (AMR) has gained national and international attention. The design and launch of national policy on antimicrobial use and resistance and action plan marked a milestone in Ghana’s commitment to control AMR. These strategies are some outcomes of getting and sustaining AMR issues prominence on government’s agenda. Understanding the agenda setting processes, policy actors involved and policy change is important as this provides insights on how and why policy actors defined and framed AMR issues to sustain its prominence despite the changing priorities of government agenda.

**Objective:**

To examine the processes of setting and sustaining AMR issues on government agenda, the policy actors involved and resulting outcomes.

**Methods:**

A qualitative study was conducted and data collected through interviewing twenty-four respondents and reviewing technical working group meeting reports and health sector documents. Data was analysed drawing on Kingdon’s agenda setting framework.

**Result:**

Members of a multisectoral technical working group (AMR platform) formed in 2011 constantly built consensus on AMR problem definition, solutions and actively engaged decision makers to mobilise support and interest. The AMR platform members sustained AMR attention and prominence on government’s agenda through the following multisectoral coordination mechanisms: (1) institutionalising AMR platform activities (2) gathering evidence, sharing findings, and supporting research (3) creating awareness and training (4) gaining and maintaining political support. The activities of the AMR platform contributed to three remarkable outcomes and these are (1) maintained network of AMR Champions, (2) design of a national policy on antimicrobial use and resistance in Ghana (1st edition) and national action plan (2017–2021), and (3) Ghana’s hosting of the second Global call to action on AMR.

**Conclusion:**

The AMR platform members as influencers concentrated their efforts to move and sustain AMR issues on government agenda. The identified multisectoral coordination mechanisms collectively contributed to agenda setting processes and policy change. The AMR platform engagements are ongoing and it is important the momentum is maintained. As multisectoral coordination and activities are vital especially for AMR ‘One Health’ approach, we hope this paper presents lessons for better understanding of how and why multisectoral groups influence national level agenda setting processes.

**Supplementary Information:**

The online version contains supplementary material available at 10.1186/s42522-021-00051-w.

## Background

Antimicrobial resistance (AMR) is a public health threat of local, national and international concern [1, 2]. Globally, AMR has gained attention at highest decision-making organs of international organization such as the World Health Organization (WHO), Food and Agriculture Organization (FAO) and the World Organization for Animal Health (OIE) [3]. At the national level, AMR is a priority government agenda and several countries have developed and implemented strategies and National Action Plans (NAP) to combat AMR [4, 5]. On the 11th of April 2018, the President of Ghana launched a national policy on antimicrobial use and resistance; and a NAP [6]. The policy provides direction and guidance for all stakeholders affected by or who use antimicrobial agents and it is implemented through the NAP [7, 8].

The design and launch of the national AMR policy and the NAP marked a milestone in Ghana’s attempt to control AMR. These strategies are some of the outcomes of AMR prominence on government’s agenda and demonstrates its’ commitment. Locally, AMR has been researched, discussed, and prioritised at health care facilities level [9–11]. However, the national level response to AMR is a result of different policy actors from the animal, human, agriculture, and environment sectors to international agencies, civil society organizations (CSOs) and academia constantly (re) defining AMR problems and (re) framing solutions and alternatives for decisions and engaging decision makers.

Government prioritized engaging stakeholders from multi disciplines, sectors and levels because of the ‘One Health’ approach. The ‘One health’ approach is *the collaborative efforts of multiple disciplines and sectors -working locally, nationally, and globally - to attain optimal health outcomes, recognizing the interconnection between people, animals, plants and our shared environment’* [12]. The ‘One health’ approach is important as countries develop strategies and systems to combat AMR [13, 14]. For instance, in the United States, federal, state and local government agencies worked collaboratively to combat AMR across sectors and one example of this collaboration is the National Antimicrobial Resistance Monitoring System [12]. Also, a quarter of countries in sub-Sahara Africa have put in place multisectoral AMR policies and NAP through the ‘One Health’ approach [5, 15, 16]. The ‘One Health’ approach promotes engaging additional multisectoral policy actors in the developing of government AMR agenda [13]. Government agenda is the list of items to which governmental officials are paying serious attention to [17].

The way policy actors define problems shapes what gets on the agenda as decision makers attention are drawn to specific problems, however, the way the problems are labelled and interpreted are equally important [17, 18]. An item’s chances of getting onto the agenda is increased if the problem definition, policy proposal and political processes are linked into a single package [17]. Setting and sustaining the issue of AMR on government agenda are critical activities because these constantly bring to the attention of decision makers the problems and possible solutions for their consideration and resolution.

Understanding the processes of setting and sustaining AMR on the government agenda and the policy actors involved is important as this provides insights on how and why AMR issues gained and sustained prominence on the government agenda and the resulting outcomes in Ghana. There is however little research on this aspect of AMR, previous studies have focused on antibiotic use and resistance policy and regulation, international focus and discourse on AMR and influence on Ghana’s decision, surveillance of AMR, leveraging donor support, AMR preparedness, antimicrobial use and resistance in food producing animals and the environment, and antibiotics stewardship [5, 6, 15, 19–22]. This research seeks to bridge the knowledge gap through describing the process of setting and sustaining AMR on governments’ agenda, the policy actors involved and the resulting outcomes.

The study presents results of analysis of policy actors involved and the processes of setting and sustaining AMR issues on government agenda and the resulting outcomes in a LMIC setting. We anticipate that these findings will provide insights to decision makers and academics interested in understanding how and why AMR items are maintained on governments’ agenda and actions taken despite the evolving nature of agenda setting as items constantly shift onto and from the agenda.

## Methods

### Study design

A longitudinal survey of the actors involved and how the AMR issues was set and sustained on the government agenda was conducted. We used a case study approach to systematically study the actors involved and the processes of AMR agenda setting and its outcomes in Ghana. Case study approach allows for the collection and analysis of in-depth information to trace policy discussions and change over time and in context [23, 24]. We defined AMR national level dialogue, action, and outcome as our case. In this study, we traced AMR national level discussions and outputs from 2011 when an AMR platform (a technical working group) was formed to understand the processes of agenda setting, the actors involved and how they defined AMR problems, framed solutions and the outcomes of their actions, inactions, and decisions over time. As noted by Sabatier, a decade is an adequate period to study a policy process change [25].

### Data collection

In-depth interviews (IDI) and document reviews were conducted from May 2019 to March 2020. Document reviews and IDI were conducted concurrently to allow for validation of the information from interviews and also allow access of relevant documents from respondents.

### Document review

AMR platform minutes of meetings from 2011 to 2019 (*n* = 21), health sector annual programme of work (2012 to 2019; *n* = 8), 2013 AMR stakeholders and situational analysis reports (n = 2), and the 2017 national policy on antimicrobial use and resistance and NAP (n = 2) were reviewed and analysed. Data on the agenda setting processes, policy actors involved, problem definition, proposed solutions, ideas and actions taken, were noted, and synthesized.

### Interviews

Individuals with experience in national level AMR discussions were purposively selected and interviewed. Scheduled IDI were conducted face to face using a semi structured guide investigating how and why the national level AMR movement (dialogue and action) started and how it has evolved over time, the ideas shared and discussed, the actors involved and the roles they played and the outcomes of these processes and interactions. The main questions were: How did the AMR agenda at national level start and how has it evolved? Which actors have been involved in the processes of setting and sustaining the agenda at national level? What are the outcomes of the AMR agenda in Ghana? See Additional file 1 for the interview guide.

Twenty-four (24) subject matter experts were interviewed (see Table 1). The respondents were selected based on their availability, knowledge, interest, influence and experience of AMR discourse and discussion at national level. The IDI were conducted in English and lasted on average 45 min. Interviews were stopped when respondents did not provide new information. Respondents were informed of the purpose of the study and consent was sought. Two respondents did not grant permission to audio record the interview therefore notes were taken and verified with the respondents.

**Table 1:** List of respondents

Sector	Organization	Number
Academia	School of Medicine and Dentistry, University of Ghana	1
	School of Biomedical & Allied Health Sciences, University of Ghana	2
	Faculty of Pharmacy, Kwame Nkrumah University of Science and Technology	1
International agencies	Food and Agriculture Organization of the United Nations	2
	World Health Organization	1
Non-governmental organization	Ghana Coalition of NGOs in Health	1
Health	Ministry of Health	6
	Food and Drugs Authority	1
	Ghana Health Service	1
	Pharmacy Council	2
Environment	Environmental Protection Agency	1
Agriculture and veterinary	Veterinary Services Directorate	1
	Fisheries Commission of Ghana	1
Professional bodies	Lady Pharmacists Association of Ghana	1
	Pharmaceutical Society of Ghana	1
Quasi government	Police Hospital	1

### Analysis

We drew on Kingdon agenda setting framework to inform our analysis and narration [17]. Kingdon argues that the processes by which items and alternatives come into prominence and the policy actors actively involved are important factors influencing agenda setting [17]. Agenda setting processes include problem definition (recognition), generation of policy proposals and political events such as change in administration [17]. The three process streams of problem, policies and politics are largely independent of one another and have their own dynamics. However, an outcome occurs when the three streams interact at certain critical times. When proposed solutions are joined to problems and ultimately joined to favourable political forces, items move up for actual actions and the outcomes could include policy change. Policy actors as entrepreneurs actively operate within these processes to influence what gets a prominent place on the government agenda [17].

The audio interviews were transcribed and read. Data from the IDI and document reviews were mapped to the research questions and further tabulated listing the policy actors involved, subgroups and committees names, objectives and setup dates, meeting agenda items and dates, decisions made, research findings shared, solutions proposed and ideas shared. The initial steps of analysis included manually coding data using the themes: actors, timelines, setting the agenda, sustaining the agenda, problem definition, generation of proposal, politics, and outcomes of the AMR movement. Further analysis involved systematically reconstructing the policy actors’ roles and the agenda setting processes of problem definition including research work conducted, solution proposed and politics and the outcomes from these processes and interactions.

### Ethical consideration

Ethical approval was sought from the University of Ghana College of Health Sciences Ethical and Protocol Review Committee with protocol identification number: CHS-Et/M.10-PI.1/2017–2018 and approved on August 3, 2018. As per the ethics, verbal and written consent were attained from all respondents before commencement of interviews. Respondent’s anonymity was maintained and protected using codes as labels.

## Results

### Setting the national level agenda for AMR

‘*There has been pockets of discussions and research on AMR in the country however, the February 2011 meeting towards developing a national policy for managing antimicrobial resistance in Ghana set the ball rolling for a national dialogue and action for AMR’* [Ministry of Health, 20th May 2019]

The Accra meetings set the momentum for AMR national level dialogue and marked the beginning of drawing national attention to AMR, mobilizing support and arousing interest of relevant stakeholders. The meetings held on the 14th and 15th February had subject experts from Action on Antibiotic Resistance (ReACT); Academia (Kwame Nkrumah University of Science and Technology (KNUST) College of Health Science, University of Ghana Medical School (UGMS) Department of Microbiology); Research institutions (Kintampo Health Research Centre (KHRC) and International Network for the Demographic Evaluation of Populations and Their Health (INDEPTH Network) and the Ministry of Health (MoH) and its agencies (Food and Drugs Authority, Ghana College of Physicians and Surgeons, Ghana Health Service (GHS) Institutional Care Division, GHS Regional Health Directorate, Office of the Chief Pharmacist, Ghana National Drugs Programme (GNDP), National Drug Information Resource Centre and Korle bu. Teaching Hospital).

‘*When we first met, the core concerns were that AMR was real, it was getting problematic and there was no national policy and framework. The discussions pointed to the fact that doing nothing will make the management of infectious diseases a nightmare’* [Academia, 15th May 2019].

During the two-day meeting different AMR problems and potential solutions were considered. AMR problems from in-country studies highlighted were multiple drug resistance to very common microbes such as *Streptococci, Salmonella*, and *E. coli* and high prevalence of *methicillin resistance staphylococcus aureus*. Additionally, the following problems were identified: absence of AMR policy and surveillance systems, inadequate education on AMR, irrational use of medicines, inadequate microbiological laboratory infrastructure and indistinct nature of AMR and inadequate microbiological laboratory infrastructure.

Participants constituted a technical working group (the Ghana AMR platform) to create awareness and mobilise the support of health professionals, academia, media, and decision makers. Professional groups and institutions with influence and interest in and affected by AMR were proposed as potential AMR platform members (see Table 2). The group agreed to present AMR challenges and potential solutions at GHS weekly meetings, the National Health Summit in April 2011, professional associations meetings and at the 2011 World Health Day celebration. Participants also agreed to advocate for AMR discussion at Drug and Therapeutic Committee reviews.

*‘ … Around February 2011, we decided to constitute ourselves into a technical working group to start discussing and dealing with issues related to AMR in Ghana. There were other people who were not on board. We identified those stakeholders and started expanding the platform. The platform was pretty expansive. It is multidisciplinary and multisectoral. These are people who are passionate and want to see action’* [Academia, 15th May 2019].

**Table 2:** List of stakeholders proposed to join the AMR working group in 2011

**Name of groupings and institutions**	
Health professional groupings e.g. Pharmaceutical Society of Ghana (PSGH) Ghana Registered Nurses Association (GRNA) Ghana Medical Association (GMA)	
Media	
Consumer Protection Groups	
Association of Representative of Ethical Pharmaceutical Industries (AREPI)	
Veterinary Service Directorate	
Christian Health Association of Ghana (CHAG)	
Law Enforcement	
Pharmaceutical Wholesalers & Importers Association	
Pharmaceutical Manufacturers Association of Ghana (PMAG)	
Pharmacy Council	
Non-Governmental Organizations (NGOs)	
Licensed Over-The-Counter Medicine Sellers	
Private health providers	
National Health Insurance Scheme (NHIS)	

### Sustaining AMR agenda

To sustain national level AMR issues on government agenda, the AMR platform members continuously defined AMR problems and framed solutions for decision makers’ consideration during their meetings and advocacy activities. AMR prominence on government agenda was sustained through the following main mechanisms: (1) institutionalising AMR platform activities (2) gathering evidence, sharing findings and supporting research (3) awareness creation, training and advocacy and (4) gaining and maintaining political support.

#### Institutionalising AMR platform activities

**Fig. 1:**
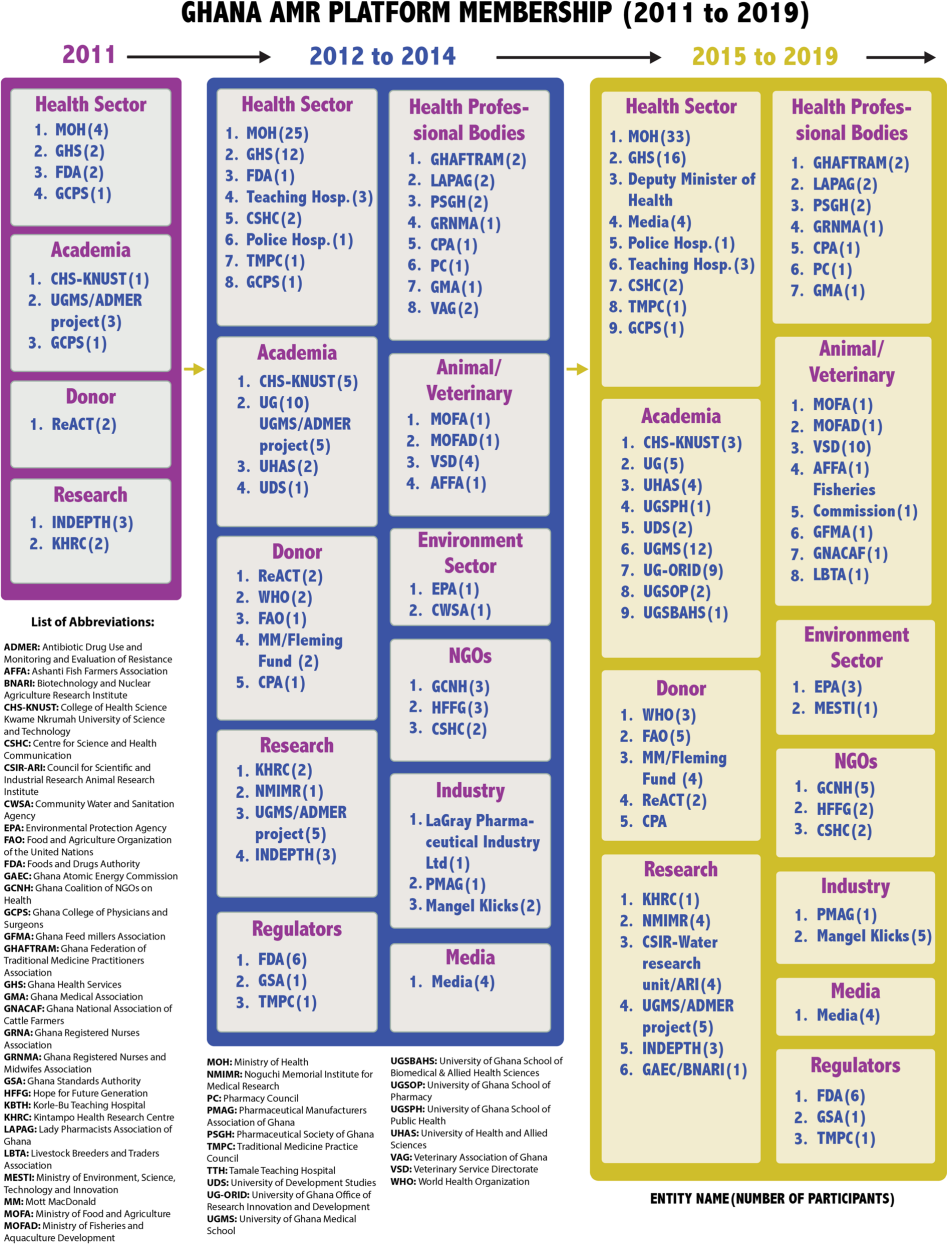
Ghana AMR platform membership (2011 to 2019)

The AMR platform activities were institutionalised when a governance structure comprising a management team and a secretariat was created by the MoH. The management team comprised of the chair, representatives from the WHO, KNUST, and head of the GNDP. The secretariat organized the AMR meetings and actively recruited new members and maintained existing membership by engaging them through regular meetings. Under the study period, the secretariat organised twenty-one (21) meetings (see supplementary Table 1). The secretariat also created a WhatsApp group titled ‘AMR Champions Platform’ in October 2016 to allow the instant sharing of information and ideas. Over time, the AMR platform membership evolved to include individuals representing veterinary, environment, animal, and food institutions with emphasis on ‘One Health’ approach. Figure 1 summarizes the increasing AMR platform membership.

‘*The AMR movement in Ghana started from the public health sector, basically the human health aspect of Antimicrobial resistance. Along the way, the animal health sector was added because we realized that significant use of antibiotics was coming from the livestock area. But with the advent of the ‘One Health’ concept: the tripartite- that is the FAO, OIE and WHO, decided to bring environment also on to the platform’* [International agency, 17th May 2019].

AMR platform members continuously created subgroups and committees to undertake specific activities and allow for active participation and consensus building. Over the study period, twenty-nine (29) groupings (subgroups, committees) were formed and dissolved when the activities were completed (see supplementary Table 2).

‘*The’ One Health approach’ reflected in the subgroups and committees formed within the platform’* [Civil Society Organization, 15th May 2019].

To support institutionalisation of AMR activities in Ghana, the MoH, Uppsala University and ReACT signed a contract in August 2012. As a result of this financial support, two coordinators were recruited for the engagement with CSOs and development of the national AMR policy respectively. Additionally, websites (http://ghndp.org/reactghana/ and http://ghndp.org/reactcso/) were developed and regularly updated by the secretariat.

#### Gathering of evidence, sharing findings and supporting research

The AMR platform became a hub for members to gather and share evidence from research. In 2013, a stakeholders analysis was conducted to identify all individuals, organizations and groups working on antimicrobial use and resistance. A stakeholders strategy was thereafter developed and used to further recruit more members. To support the design of the AMR policy, situational analysis of the following was conducted in 2013: antimicrobial resistance trends, use of antimicrobial in veterinary and aquaculture, rational use of medicines, infection prevention and control, laboratory diagnostics and protocol, national surveillance systems for antimicrobial, regulation and enforcement, antimicrobial manufacturing, distribution and use and legislative and legal framework.

*‘At that time, we thought we needed some baseline data to really see what was happening in the country’ [Ghana Health Service, 31st May 2019].*

Additionally, surveys were conducted by members between December 2012 and March 2013 to assess the AMR knowledge, attitude, and practice by health related CSOs and health professionals.

*‘Hitherto we hadn’t analysed the enormity of the AMR problem. But thanks to the movement, we now have an idea of what we are dealing with. Using the various civil society organizations, we are letting people know that you don’t use antimicrobials anyhow’* [Academia, 15th May 2019].

Members regularly shared AMR decisions and activities undertaken in their individual organizations. Additionally, representatives updated members on the following projects: Antibiotic Drug use, Monitoring and Evaluation of Resistance (ADMER), Monitoring Antimicrobial Resistance in Hospital Laboratories (MARHLAB), Healthcare associated infection (HAI), Tricycle *E. coli*, University of Health and Allied Science Scottish-West Africa partnership to fight Antibiotic Resistance (UHAS SWAB), Fleming funded and the Global AMR surveillance systems (GLASS).

‘*The AMR platform became a very important source of knowledge for health professionals because research findings were constantly shared and innovative ideas discussed*.’ [Professional body, 20th May 2019].

Research, training, and funding opportunities were also discussed and members encouraged to apply. Notable were the Fleming funding and fellowship, and the Structured Operational Research Training Initiative (SORT IT) research. On 15th February 2017, a team of the UK Fleming fund country grant mission briefed members on the aims and expectation of the Fleming Fund.

*‘People are interested in Ghana’s AMR activities setting the foundation for FAO support and the Fleming fund. This was not just an issue of FAO Ghana but the support came right from the headquarters. Ghana was selected as one of the seven countries offered financial support for AMR work and this is all because of the work done in Ghana’* [International agency, 17th May 2019].

#### AMR awareness creation, training and advocacy

AMR platform members advocated for AMR issues and participated in sensitization programmes to educate the general public and in July 2014 designed and aired an AMR documentary on national television (MultiTV). In addition, training programmes were organised and by October 2014 ninety-one (91) members of CSOs were trained. Community leaders including Queen Mothers and representatives from municipal health directorates in the Central and Western regions were sensitized on AMR. The guide used for the training titled ‘Fighting Antibiotic Resistance in Ghana: Manual for Training Civil Society Organisations’ was developed by the platform members and reviewed by two independent reviewers.

‘*One can say that the capacity of various CSOs has been enhanced because of the advocacy and training activities of the AMR platform. Now we are AMR champions and involved in research generating evidence as well as influencing policy’*[Civil Society Organization,15th May 2019].

AMR platform members, also, participated in international meetings. For example, a seven-member delegation from the AMR platform and led by the deputy Minister of Health attended the 1st Global Forum on Antibiotic Resistance conference (3–5 October 2011) in New Delhi, India. The Minister made a presentation highlighting Ghana’s efforts and commitment towards tacking AMR. He was also signatory on a global ministerial communique to preserve the power of antibiotics for future generations. Thereafter, three members of the platform participated in a ReACT CSO project management and implementation meeting in Penang, Malaysia (17–18 February 2012) and a ReACT consultative meeting in Uppsala Sweden (21–22 February 2012). Additionally, two members represented the AMR platform at the ReACT CSO project workshop (November 2012) in Cuenca, Ecuador. The platform chair participated in the 67th and 68th World Health Assembly, the Dag Hammarskjöld Foundation forum in Sweden and ministerial forum in the Netherlands to share Ghana’s efforts to tackle AMR.

*‘We learnt from other country examples. We also had the opportunity to further discuss AMR in-country findings at a conference in Istanbul, Turkey. The AMR discussions were kept alive and translated into a national policy’* [Academia 15th May 2019].

#### Gaining and maintaining political support

Throughout the study period, the AMR platform engaged with national politicians and key decision makers. The Ministers of Health were constantly briefed of the activities of the AMR platform. On 10th July 2014, the chair of the Parliamentary Select Committee on Health expressed his interest and governments’ commitment to tackle AMR issues after he was briefed on the efforts of the AMR platform. Members also briefed the National Development Planning Commission and contributed to the design of AMR indicators for the country’s long-term plan. Additionally, to demonstrate further support, the GHS Director General participated in the 29 June 2017 meeting. AMR platform members engaged the wider leadership of the health sector as they presented AMR problems and potential solutions at the Health Sector working meetings and the annual Health Summit. As a result, antimicrobial use and resistance was captured as policy strategy in the 2018 health sector Aide Memoire. On October 2, 2019 findings from Tricycle *E.coli* study was disseminated at the health sector working group meeting.

*‘Presenting AMR issues at the health summit and health sector working group meeting were very important because these are the decision-making spaces where strategies are firmed up for the health sector plans and implementation’* [Ministry of Health, 11th June 2019].

In August 2016, the members briefed the Chief Directors and Ministers of the Ministries of Fisheries, Agriculture, Environment Science Technology and Innovation on the design and different aspects of the AMR policy. During the October 2016 validation workshop for the NAP, the Deputy Minister of Food and Agriculture, and the Senior Policy Advisor to the Chief Veterinary Officer at the FAO Head Quarters, pledged their support to AMR activities in country.

Over time, some global AMR actors participated in the AMR platform meetings. For instance, the ReACT global coordinator, global network focal person and a representative from the Africa office participated in AMR platform meetings on 10th May 2012, 11 July 2013 and 23 March 2015 respectively. Prof Otto Cars, founder of ReACT, also participated in the 21 November 2018 meeting to gain a first-hand experience about Ghana’s AMR platform and share knowledge.

### Outcomes of sustained AMR agenda

AMR platform members actions resulted in three remarkable outcomes: (1) maintained network of AMR Champions, (2) design and launch of a national policy on antimicrobial use and resistance in Ghana (1st edition) and its accompanying NAP (2017–2021) and (3) Ghana’s hosting of the second Global call to action on AMR.

One, a network of AMR champions formed and maintained as a result of the platform members regular communications of AMR problems, exchange of ideas, policy proposals, research findings and funding opportunities. This allowed for a pattern of collective actions among public and private actors in the fight against AMR.

‘*The AMR platform over time has become a space where decisions are taken. Also the membership increased and it keeps growing because people are showing interest and are passionate about AMR issue. We started with a few people-about 20 and now the platform has about 75 members ’* [Ministry of Health, 15th May 2019].

**Fig. 2:**
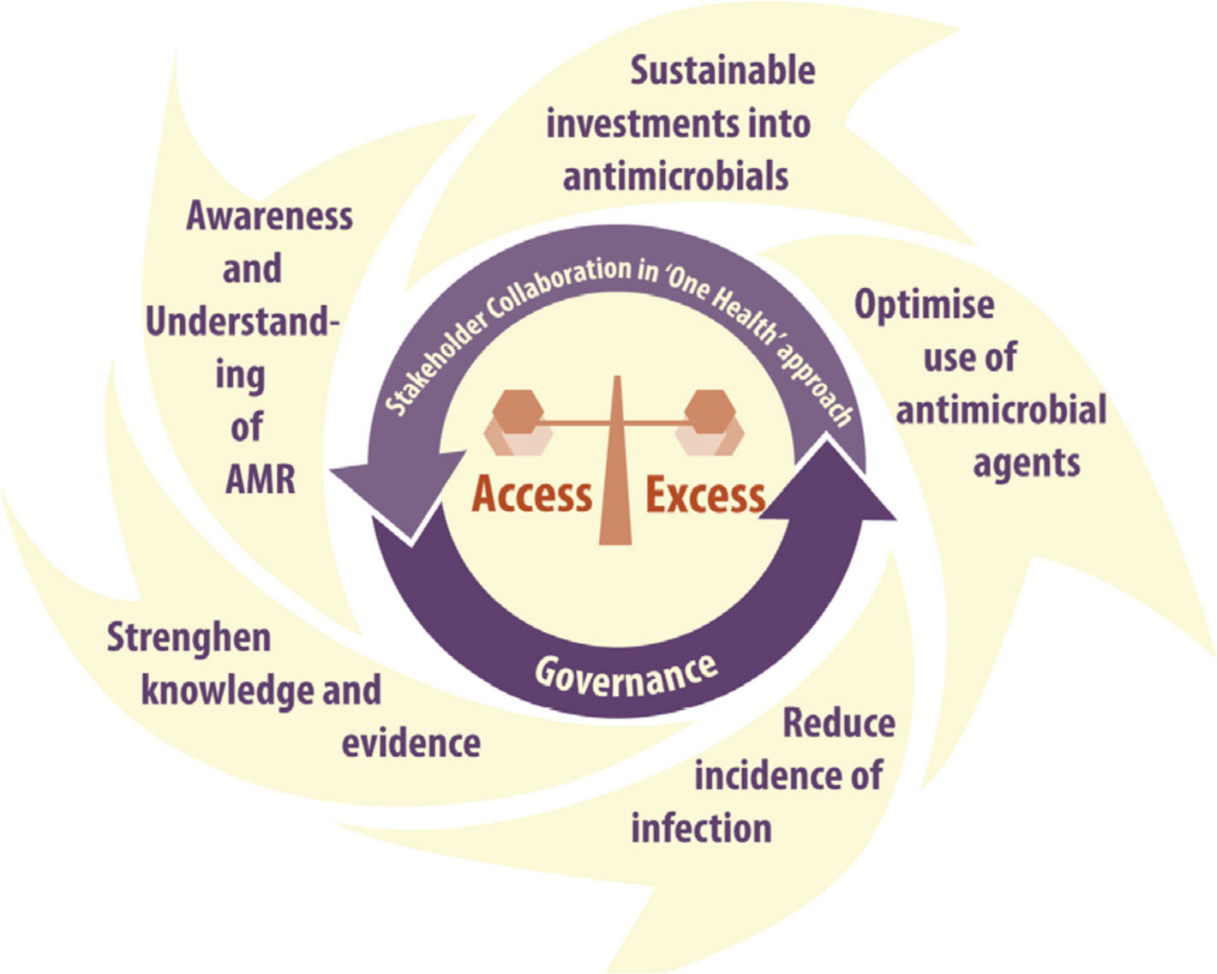
Conceptual framework for AMR interventions in Ghana. Source: Policy on Antimicrobial use and resistance 1st edition (2017)

Two, a national policy designed to improve awareness of AMR through effective communication, education and training and strengthen existing knowledge base through surveillance and laboratory services. As well as reduce incidence of infection through infection prevention measures and ensure a balance between access and excess to preserve antimicrobials [7]. Figure 2 depicts the conceptual framework for AMR interventions in Ghana [7]. The NAP is designed to promote combined efforts and resources, and provide timelines for strategic plans, operational plan, process indicator matrix, budget, monitoring and evaluation framework [8].

*‘Ghana was the first country in Sub-Saharan Africa to launch its national AMR policy and action plan. So Ghana is seen as a leader when it comes to the issue of AMR and this is an output from the AMR platform activities.’*[Professional body, 20th May 2019].

The government of Ghana, Swedish International Development, the World Health Organization, the ADMER project, Ghana, and the Food and Agriculture Organization (FAO), Ghana office provided financial and technical support for the design of the AMR policy and NAP.

‘*Ghana has put together a policy and is no longer confused as to what it wants to do concerning AMR. Ghana is very clear on its intentions on AMR and what interventions should be taken. And that is a huge achievement’* [Ministry of Health, 22nd May 2019].

Three, the government of Ghana in partnership with the Inter-Agency Coordination Group on Antimicrobial Resistance organised the second call to action on AMR in Ghana in November 2018. The conference brought together diverse stakeholders.

‘*Ghana has benefited so much since the AMR movement started. The Second Global call to Action conference was held in Ghana on the back of Ghana’s achievements towards curbing AMR menace. Even though we have chalked some success we need to sustain the discussions and consolidate the work done thus far’* [Academia,15th May 2019].

## Discussion

The case of AMR national level dialogue and activities illustrates the collective action by stakeholders (champions) in defining and framing AMR problems and solutions and building consensus to set and sustain AMR issues on government agendas. These champions were from diverse sectors, disciplines and jurisdictions with a common goal of highlighting the challenges of AMR and proposing potential solutions. The collective action of these stakeholders depicted the multisectoral approach of the AMR platform. Initially, stakeholders were mainly from the health sector and this gradually changed to include others from animal health, veterinary, husbandry, agriculture, aquaculture and environment were included in line with the ‘One Health’ approach. As a result, the framing of AMR problems and solutions moved beyond health and framed as a threat to all sectors including animal, human, plants and environment. The ‘One Health’ approach later adopted by the AMR platform reflected the Global Health Security Agenda AMR action package which promotes multisectoral engagement and collaboration [26].

Members of the AMR platform from government ministries, research institutions, academia, professional bodies, CSOs and international agencies were actively involved, passionate and participated in discussions to move AMR issues onto government agenda, attract and maintain their interest and develop policies and national action plans for implementation. This is similar to previous works in some selected LMIC settings, where collective actions through multisectoral groups resulted in outcomes such as AMR national action plans and policies [5, 15, 27].

The AMR platform activities thrived in an institutionalised venue managed by the AMR secretariat and management team (governance structure). This venue served as bureaucratic and public decision making space where decisions taken by members were acted upon by responsible organization and sector [28]. Government ministries together with other AMR platform members discussed and took decisions that had effects in their various bureaucratic settings. AMR platform members moved decisions and inputs from the AMR platform to implement within their respective ministries and bureaucratic settings. For example, members briefed their respective Ministers of actions, decisions and activities of the AMR platform and incorporated inputs during the development of the national policy on antimicrobial use and resistance and action plan. Additionally, members engaged within the public venue where they educated the general public and professional associations on the menace of AMR and appropriate use.

AMR platform membership was open as members actively contributed to the development of the national AMR policy and the NAP. This is however not the case for other policy making venues within the Ghanaian setting. For example, policy decision making in the Parliament and within the health sector business meetings are restricted to individuals with the mandate to participate although the policies are of national interest [29, 30]. The AMR platform allowed participation from all sectors, disciplines and jurisdictions through the gathering of evidence, sharing of findings, ideas and proposals and advocacy. Though problem definition can be based on individual understanding and interpretation of what the issue is, AMR platform members were unified and built consensus on what to consider as AMR problems and the potential solutions [17]. The way AMR issues are framed for attention is important for political attention and action [31]. Members not only framed and highlighted AMR problems and potential solutions but also arouse political and public interest with alarming indicators and findings from research. AMR is still a global issue with ongoing international discourse and this is reflected in donor support for AMR activities [6]. Getting politicians, policymakers and the public to appreciate and pay attention was a major political gain [17, 31].

The AMR platform as a policy community floated ideas and solutions on strategies to improve AMR awareness, strengthen knowledge and evidence base, reduce incidence of infection, optimise the use of antimicrobial agents and develop an economic case for sustainable investments in new medicines, diagnostic tools and other interventions [17]. These strategies are captured in the national AMR policy and action plan [7, 8]. These ideas and solutions created opportunities for actions and outcomes [17]. The AMR platform outcomes were not instant. These were results of several years of multisectoral engagement, consensus building, resilient governance structure and concerted discussions of issues and ideas. For example, the need for a national AMR policy was discussed in 2011, however, the policy was finalised in 2017 and launched by the President in April 2018. This may seem long but building a robust policy community working from different sectors and disciplines within a hybrid institutionalised venue can be a challenge. As noted by Joshi et al., coordinating and working across sectors and creating ownership require time and therefore the need to identify best practice on how to balance individual priorities to a common goal in a timely manner [27].

The policy change initiated by the AMR platform were driven by the definitions and framing of AMR problems and solutions, this was further advocated by politicians and decision makers. Policy change is complex and multifaceted, and as politicians and decision makers process evidence and solution disproportionately, they choose why and how to act and sustain AMR agenda [32]. The policy change and activities of the AMR platform are laudable and can serve as lessons for other settings. It is however important to continue with the momentum. The 5 year NAP ends in 2021, and as AMR platform members and other relevant ministries and sectors work on the next strategic plans, there is the need to evaluate the 2017–2021 NAP to provide guidance. We recommend further research to evaluate the 2017–2021 NAP with emphases on resources mobilization and funding sources for implementation.

### Study limitation

This study has a number of limitations. There are potential recall biases as key informants addressed how and why national level AMR movement started and evolved. Additionally, decisions started in AMR platforms meeting reports were recorded as collective decisions masking the details of who said what and how it was received. To mitigate these challenges, we used varied data sources to reconstruct the AMR agenda setting processes and outcomes.

## Conclusion

These multisectoral coordination mechanisms: institutionalisation of AMR platform activities, gathering and sharing of evidence, awareness creation and advocacy and gaining, and maintaining political support collectively contributed to sustaining AMR issues on government agenda. The AMR platform activities and engagements are ongoing and it is important the momentum is maintained and intensified. As multisectoral coordination and activities are vital especially for AMR ‘One Health’ approach, we hope this study documents lessons that will improve understanding of justification and functioning of multisectoral groups in influencing national level agenda setting processes.

## Supplementary Information

**Additional file 1.** AMR Movement Semi- Structured Interview Guide.

**Additional file 2.** Supplementary Table 1: AMR platform meeting dates and agenda (2011-2019).

**Additional file 3.** Supplementary Table 2: Subgroupings names, objectives and setup dates.

## Data Availability

The datasets generated and analyzed during the current study are not publicly available due to the respondents’ consent to use the data for this research specifically. Data can be available upon reasonable request to the corresponding author.

## Abbreviations

ADMER: Antibiotic Drug use and Monitoring and Evaluation of Resistance
AFFA: Ashanti Fish Farmers association
AMR: Antimicrobial Resistance
BNARI: Biotechnology and Nuclear Agriculture Research Institute
CHS-KNUST: College of Health Science Kwame Nkrumah University of Science and Technology
CSHC: Centre for Science and Health Communication
CSIR-ARI: Council for Scientific and Industrial Research Animal Research Institute
CSO: Civil Society Organization
CWSA: Community Water and Sanitation Agency
EPA: Environmental Protection Agency
FAO: Food and Agriculture Oorganization
FDA: Foods and Drugs Authority
GAEC: Ghana Atomic Energy Commission
GCNH: Ghana Coalition of NGOs on Health
GCPS: Ghana College of Physicians and Surgeons
GFMA: Ghana Feed Millers Association
GHAFTRAM: Ghana Federation of Traditional Medicine Practitioners Association
GHS: Ghana Health Service
GMA: Ghana Medical Association
GNACAF: Ghana National Association of Cattle Farmers
GNDP: Ghana National Drugs Programme
GRNMA: Ghana Registered Nurses and Midwives Association
GSA: Ghana Standards Authority
HFFG: Hope For Future Generation
KBTH: Korle bu. Teaching Hospital
KHRC: Kintampo Health Research Centre
KNUST: Kwame Nkrumah University of Science and Technology
LAPAG: Lady Pharmacists Association of Ghana
LBTA: Livestock Breeders and Traders Association
LMIC: Low Middle Income Country
MESTI: Ministry of Environment, Science, Technology and Innovation
MM: Mott MacDonald
MOFA: Ministry of Food and Agriculture
MOFAD: Ministry of Fisheries and Aquaculture Development
MoH: Ministry of Health
NAP: National Action Plan
NMIMR: Noguchi Memorial Institute for Medical Research
OIE: World Organization for Animal Health
PC: Pharmacy Council
PMAG: Pharmaceutical Manufacturers Association of Ghana
PSGH: Pharmaceutical Society of Ghana
ReACT: Action on Antibiotic Resistance
TMPC: Traditional Medicine Practice Council
TTH: Tamale Teaching Hospital
UDS: University of Development Studies
UG-ORID: University of Ghana Office of Research Innovation and Development
UGMS: University of Ghana Medical School
UGSBAHS: University of Ghana School of Biomedical & Allied Health Sciences
UGSOP: University of Ghana School of Pharmacy
UGSPH: University of Ghana School of Public Health
UHAS: University of Health and Allied Sciences
VAG: Veterinary Association of Ghana
VSD: Veterinary Service Directorate
WHO: World Health Organization
