# Supplementary material for: Antimicrobial resistance national level dialogue and action in Ghana: setting and sustaining the agenda and outcomes
Source: One Health Outlook. 2021 Oct 19;3:18. doi: 10.1186/s42522-021-00051-w (PMC8524845; doi:10.1186/s42522-021-00051-w)
Supplement: Supplementary file 1 — Additional file 1. AMR Movement Semi- Structured Interview Guide. [file 42522_2021_51_MOESM1_ESM.docx]

**AMR Movement Semi- Structured Interview Guide**

1. To the best of your knowledge how did the discussions around AMR start at national level in Ghana?
   1. What are some of the issues discussed? What decisions were taken and why?
   2. How were AMR issues defined and what solutions were proposed?
2. How have these discussions evolved over time and why?
   1. In your opinion what discussion points remained over time and why?
   2. What ideas, evidence and solutions were shared during these discussions and why?
   3. What are the current discussions on AMR in Ghana?
3. Who were the main actors involved in these discussions and what were their concerns?
   1. What institutions, grouping did these actors represent and what roles did they play?
   2. Which other actors were involved in the AMR discussions over time and why?
   3. Which actors are currently involved in the discussions at national level and what ideas, evidence and solutions are shared?
4. In your opinion, how has Ghana benefited from the AMR movement?
   1. Kindly list some of these benefits and why are these significant?
