# Supplementary material for: Antimicrobial resistance national level dialogue and action in Ghana: setting and sustaining the agenda and outcomes
Source: One Health Outlook. 2021 Oct 19;3:18. doi: 10.1186/s42522-021-00051-w (PMC8524845; doi:10.1186/s42522-021-00051-w)
Supplement: Supplementary file 2 — Additional file 2. Supplementary Table 1: AMR platform meeting dates and agenda (2011-2019). [file 42522_2021_51_MOESM2_ESM.docx]

Supplementary Table 1: AMR platform meeting dates and agenda (2011-2019)

| **Date** | **Meeting Agenda** | |
| --- | --- | --- |
| 14 &15 February 2011 | - Overview of the AMR problem and the principal management options - Data on AMR in Ghana - Presentation on the Kintampo story | - Moving towards national policy for management of AMR – experiences from Thailand - General overview of different AMR management options - The ADMER Project |
| 18 August 2011 | - Development of AMR policy - Collaboration with stakeholders for the establishment of surveillance centers | - 1st Stakeholders’ conference (2012) with broad participation, cross-learning - DTC peer review to put AMR on agenda - Proposal to SIDA |
| 8 February 2012 | - CSO Project on AMR - Ghana-ReAct Collaboration | - Debrief from New-Delhi forum (1^st^ Global Forum on Antibiotic Resistance) - Ghana – Global Antibiotic Resistance Partnership Collaboration |
| 10 May 2012 | - Debrief from Penang and ReAct Uppsala Meetings - Update Ghana – Global Antibiotic Resistance Partnership Collaboration | - CSO Project implementation - Working Group Sub-Committee Reports |
| 23 April 2013 | - Debriefs – Cuenca CSO workshop, ReAct Projects & Policy Meetings - CSO Project Update – Knowledge Attitude Belief and Practice (KABP) presentation | - Monitoring Antibiotic Resistance in Hospital Laboratories (MARHLAB) - Policy Development Process - KABP – Health Professionals |
| 11 July 2013 | - KABP Health Professionals – Research Group - Monitoring Antibiotic Resistance in Hospital Laboratories, Way forward - Updates on ReAct projects - Proposal on AMR 2003 study | - ADMER project updates - Policy Development Process (Updates) - AMR in Veterinary |
| 2 April 2014 | - KABP Health Professionals – Research Group - Updates on ReAct projects – CSO and National Policy on Antimicrobial Resistance (NPAR) - Proposal on AMR 2003 study | - ADMER project updates - Policy Development Process (Updates) - AMR in Veterinary (Updates) |
| 10 July 2014 | - Travel updates and reports - Updates on NPAR project | - Update on CSO project |
| 21 October 2014 | - Updates CSO project - Updates form NPAR project - AMR Policy Process, Situational Analysis Report | - KABP Health Professionals, Final report - Final Report from ADMER - Way forward for AMR actions in Ghana |
| 25 April 2014 | - Fleming Fund country Grant updates and next steps - Knowledge repository for Fleming Fund surveillance project/Mainstreaming AMR into MDAs | - UHAS SWAB project overview - Updates from FAO/MOFA - Overview-Commonwealth Partnerships for Antimicrobial Scheme (CwPAMS) - Tricycle E.coli project |
| 30 August 2016 | - Proposals from the food, agriculture and fisheries sectors into the AMR Policy for Ghana | - Discussions & contributions and adoption of presentation - Presentation on National Action Plan |
| 27 & 28 October 2016 | - AMR national one health workshop for the national action for Ghana: Validation of the national action plan - Presentation on the Draft AMR Policy | - AMR Global Perspective - Remarks by Food and Agricultural Organization, World Health Organization, Ministry of Health, Ministry of Food and Agricultural |
| 15 February 2017 | - Update on the National Action Plan - UK Fleming Country Grant Mission | - National Surveillance of Antimicrobial Resistance in Ghana: mission - Update from the Joint External Evaluation (JEE) on AMR |
| 13 April 2017 | - Update of the Fleming fund | - National Action Plan |
| 29 June 2017 | - Joint stakeholder meeting of the antimicrobial resistance platform on the national action plan of the AMR - Update of the Fleming fund | - Overview of NAP - Message from Director – General of Ghana Health Service - Group discussion session and validation |
| 21 November 2018 | - AMR – Drug Development- Omadacycline Updates from Veterinary Service Directorate - Global AMR Surveillance System (GLASS) - Updates on Fleming Fund activities - Updates from Tricycle E. coli project | - Report from the mid-term review of Healthcare Associated Infection (HAI) - Surgical Site Infections (SSIs) surveillance - Antimicrobial resistant pathogens from blood culture - Quality of antimicrobial agents |
| 25 April 2019 | - Fleming Fund country Grant updates and next steps - Knowledge repository for Fleming Fund surveillance project/Mainstreaming AMR into MDAs | - UHAS SWAB project overview - Updates from FAO/MOFA- AMU monitoring systems - Overview-Commonwealth Partnerships for Antimicrobial Scheme (CwPAMS) - Tricycle E.coli project |
| 18 June 2019 | - Fleming Fund AMR Surveillance sub-committee updates and next steps - Tricycle *E*.*coli* project - Presentation on Infection Prevention Control practice in GHS - FDA presentation on Quality of antibiotics on the market | - Structured Operational Research and Training Initiative (SORT IT) project overview - Access to antibiotics in rural Ghana: regulatory and community demands - Updates from FAO/MOFA - GLASS update - Overview-Commonwealth Partnerships for Antimicrobial Scheme (CwPAMS) |
| 21 August 2019 | - Statements by agencies, ministry of health and tripartite Overview of AMR in Ghana – Implications in one health - Fleming Fund Laboratory Surveillance project updates | - Overview and findings – Tricycle Project - Ministries Agency Responsibilities on NAP - Draft TORs for Inter-Ministerial Committee |
| 2 October 2019 | - Fleming Fund Project updates - SORT IT Application outcome - Updates from FAO - Kintampo presentation | - Presentation on E coli in animal feed - Updates from MoFA/VSD - Updates Commonwealth Partnerships for Antimicrobial Stewardship program |
| 20 December 2019 | - Fleming Fund Project Updates - Summary Presentation from Secretariat - FDA Presentation | - SORT IT Fellows presentation - Mapping antimicrobial resistance and antimicrobial use partnership (MAAP) Presentation and open forum to discuss implementation |
