# Supplementary material for: Antimicrobial resistance national level dialogue and action in Ghana: setting and sustaining the agenda and outcomes
Source: One Health Outlook. 2021 Oct 19;3:18. doi: 10.1186/s42522-021-00051-w (PMC8524845; doi:10.1186/s42522-021-00051-w)
Supplement: Supplementary file 3 — Additional file 3. Supplementary Table 2: Subgroupings names, objectives and setup dates. [file 42522_2021_51_MOESM3_ESM.docx]

Supplementary Table 2: Subgroupings names, objectives and setup dates

| **Subgroupings name** | **Objectives** | **Setup date** |
| --- | --- | --- |
| Conference Planning Committee | Committee to propose the theme, venue and protocol for stakeholders conferences | 18 August 2011 |
| Surveillance Group | To undertake needs assessment of the research centres involved (Navrongo, Kintampo, Hohoe, Dodowa, Agogo) to determine their capacity to undertake culture, sensitivity and surveillance | 8 February 2012 |
| Knowledge Attitudes Belief and Practice (KABP) group | Draft a proposal on knowledge, attitude, behaviour and practices on AMR | 8 February 2012 |
| Infection Control Subcommittee | The committee was tasked to provide a summarized version of the infection control policy for inputting into the AMR policy framework | 10 May 2012 |
| Thematic groups:  Rational use of Medicine  Strengthening Disease Control Programmes  Infection Prevention and Control  Waste Management  Laboratory Diagnostics and Protocol Requirements  National Surveillance Systems  Antimicrobial Manufacture, Distribution and Use | To provide inputs for the development of the National AMR policy | 23 April 2013 |
| Terms of Reference Group | To develop broad terms of reference for the AMR policy process and to define membership with clear work plan and timelines: | 23 April 2013 |
| Subgroup on Food and Agriculture | To support the AMR policy Technical Working on Food and Agriculture with inputs for the AMR policy | 30 August 2016 |
| Advocacy Team | To present the AMR policy summary to Chief Directors of the ministries of Fisheries, Agriculture, Environment Science Technology and Innovation and the Ministry of Health | 30 August 2016 |
| Education and Awareness  Surveillance and research  Antimicrobial Use  Hygiene and Infection prevention and control  Investment | Subgroupings revised with increasing membership to promote the work of the platform and the AMR policy | 30 August 2016 |
| Members regrouped into teams namely: Communication, Surveillance, Infection control, Economic and Use of Antimicrobials. | To validate different session of the draft AMR policy | 27 & 28 October 2016 |
| Surveillance Technical Working group  Advocacy Technical Working group  Infection prevention and control Technical Working group  Policy Technical Working group  Stewardship Technical Working group  Research and development Technical Working group | Recreated subgroupings so as to include new members | 25^th^ April 2019 |
